# Thermogravimetric analysis and kinetic modeling of the pyrolysis of different biomass types by means of model-fitting, model-free and network modeling approaches

**DOI:** 10.1007/s10973-023-12868-w

**Published:** 2024-03-21

**Authors:** Olivier Fischer, Romain Lemaire, Ammar Bensakhria

**Affiliations:** 1 TFT Laboratory, Department of Mechanical Engineering, École de Technologie Supérieure, Montreal, QC H3C 1K3 Canada; 2 ESCOM, TIMR, Centre de Recherche Royallieu, Université de Technologie de Compiègne, CS 60 319, 60203 Compiègne Cedex, France

**Keywords:** Biomass, Thermogravimetric analysis, Kinetic modeling, Model-fitting, Model-free methods, Network model

## Abstract

This work aims at comparing the ability of 7 modeling approaches to simulate the pyrolysis kinetics of spruce wood, wheat straw, swine manure, miscanthus and switchgrass. Measurements were taken using a thermogravimetric analyzer (TGA) with 4 heating rates comprised between 5 and 30 K min^−1^. The obtained results were processed using 3 isoconversional methods (Kissinger–Akahira–Sunose (KAS), Ozawa–Flynn–Wall (OFW) and Friedman), 1-step and 3-step Kissinger models, as well as an advanced fitting method recently proposed by Bondarchuk et al. [1] (Molecules 28:424, 2023, 10.3390/molecules28010424). Seventeen reaction models were considered to derive rate constant parameters, which were used to simulate the variation of the fuel conversion degree $\alpha$ as a function of the temperature $T$. To complement this benchmarking analysis of the modeling approaches commonly used to simulate biomass pyrolysis, a network model, the bio-CPD (chemical percolation devolatilization), was additionally considered. The suitability of each model was assessed by computing the root-mean-square deviation between simulated and measured $\alpha =f(T)$ profiles. As highlights, the model-free methods were found to accurately reproduce experimental results. The agreement between simulated and measured data was found to be higher with the Friedman model, followed by the KAS, FWO, 3-step, and 1-step Kissinger models. As for the bio-CPD, it failed to predict measured data as well as the above-listed models. To conclude, although it was less efficient than the Friedman, KAS or OFW models, the fitting approach from Bondarchuk et al. [1] (Molecules 28:424, 2023, 10.3390/molecules28010424) still led to satisfactory results, while having the advantage of not requiring the selection of a reaction model a priori.

## Introduction

Interest in the use of renewable and carbon-neutral energy resources, such as biomass, continues to grow, given that the challenges related to increasing energy demand and the depletion of fossil fuel resources must be tackled, even while addressing the overarching need to reduce greenhouse gas (GHG) emissions. In this context, pyrolysis has proven to be a promising route to convert raw biomass into a wide variety of high-value biochemicals and biofuels [2–4]. More specifically, pyrolysis refers to the conversion of biomass by the action of heat in an inert atmosphere as defined in [2–4] among others. During the biomass heating, the biopolymers composing it typically decompose into products formed in three states. These consist of a carbon-rich solid residue called biochar, a condensable vapor fraction made up of a complex mixture of water and organic species which corresponds to the so-called bio-oil, and a non-condensable gaseous phase composed of CO, CO_2_, H_2_, CH_4_, etc., also referred to as syngas. While biochar is of interest for soil amendment and GHG capture among other things [5, 6], syngas and bio-oil are more specifically devoted for use as substitute fuels for boiler and engine applications [2].

In expanding the application of pyrolysis to produce upgraded biochemicals as well as biofuels having a higher energy density as compared to raw biomass, a key challenge is to develop the computational codes required to design and/or optimize the functioning of pyrolizers [7]. Doing so implies gaining a fundamental knowledge of the mechanisms underlying the thermal conversion of solid fuels. For over a half century now, major progress in the field has been achieved as reported in various reviews (see [2–4] as examples and references therein). Nevertheless, and due to the wide variety of biomass in existence and to the significant heterogeneity that can be observed within a given feedstock type, current modeling tools are still unable to fully capture the complexity of biomass decomposition, which is influenced by numerous operating factors, including the temperature, the heating rate, etc. [8]. Continuous effort is therefore being investing to experimentally characterize the thermal degradation of different kinds of biomass with the aim of inferring kinetic parameters to be embedded within current pyrolysis models in order to improve their predictive ability. To that end, widely varying methods (see below) can be used, noting that their parameterization against trusted data still prompts the need for complementary analyses conducted using reference analytical devices such as thermogravimetric analyzers (TGA).

Among the models commonly used to simulate the thermal decomposition of solid fuels including biomass (see [8] and references therein), one can mention the global schemes based on single or multiple parallel and competitive reactions, the distributed activation energy methods and the network modeling approaches, including the functional group depolymerization vaporization crosslinking (FG-DVC), FLASCHAIN and chemical percolation devolatilization (CPD) models. These simulation tools can be roughly classified into different categories, depending on whether they aim at simulating either the mass loss rate of the fuel, the distribution of the pyrolytic products, or both. That being said, and in the context of TGA-based kinetic analyses, model-fitting (e.g., Coats–Redfern, Šatava–Šesták, etc.) and model-free methods (e.g., Kissinger–Akahira–Sunose (KAS), Ozawa–Flynn–Wall (OFW), Friedman, Kissinger, etc.), continue to be extensively used in estimating kinetic parameters (see [9–15] for instance). The use of a model-free approach is particularly recommended as a trustworthy way of obtaining reliable and consistent kinetic information as it allows directly inferring activation energies without the need for any initial assumption regarding the reaction model [16]. In a recent work, [1] still proposed a so-called advanced fitting method for identifying kinetic triplets using the integral method applied to a solid-state reaction model based on a modified Arrhenius equation. The advantage of this approach is that it does not require any a priori assumption regarding the reaction mechanism. The latter is indeed expressed based on the Šesták–Berggren equation [17], whose parameters are directly calculated through an optimization procedure aimed at minimizing the scatter in the frequency factors calculated for different ranges of temperatures and conversion degrees derived from a data table built upon the results from the thermogravimetric experiments (see Sect. “Fitting method”).

The above literature survey thus shows that numerous modeling approaches can be considered to simulate biomass pyrolysis and/or infer kinetic triplets through the processing of experimentally monitored mass loss profiles. The so-assessed parameters are very sensitive to the methods used, however, as pointed out in [9]. There is therefore a crucial need to compare the ability of the main models commonly used in the literature to properly capture the kinetics at play during the pyrolysis of a wide variety of biomass types so as to obtain consistent and reliable rate constant parameters. Although different reviews previously focused on the kinetic modeling of the thermal degradation of biomass (see [8, 18, 19] as examples), the assessment of the relative ability of existing models to infer suitable kinetic triplets based on a given set of experimental data was out of the scope of these papers. To the best of the authors’ knowledge, one of the few works aimed at evaluating modeling approaches to determine kinetic parameters for biomass pyrolysis by TGA was proposed by [9]. In this very thorough analysis, the authors compared the kinetic parameters of beechwood and flax shives pyrolysis derived using Kissinger, KAS, Friedman and based modeling approaches. The obtained results then illustrated large discrepancies for the same experimental results even for pure pseudo-components while concluding that the Kissinger method was considered the most adapted for the determination of kinetic parameters. Only two different feedstocks (in addition to pure hemicellulose, cellulose and lignin) were tested therein, however, while no phenomenological model was evaluated.

To address these lacks and thus complement the elaborate study by [9], the present work aims at comparing 7 different modeling approaches used to simulate the pyrolysis of 5 biomass types. These consist of 5 model-free methods, including 3 isoconversional models (Kissinger–Akahira–Sunose (KAS), Ozawa–Flynn–Wall (OFW) and Friedman) together with 1-step and 3-step Kissinger modeling approaches. In addition, the advanced fitting method recently proposed by [1] as well as the bio-CPD [20] were additionally implemented to assess their predictive capability as compared to the results obtained from the implementation of the above-listed model-free approaches. While many kinetic modeling studies consider 2 or 3 methods to estimate the rate constant parameters of the studied samples (see [10–15] as examples), studies based on the use of 7 different models including model-free, model-fitting and phenomenological approaches are quite rare. Furthermore, and in addition to the use of the bio-CPD model, which has seldom been considered within the context of benchmarking analyses, the present work also represents the first attempt at comparing the effectiveness of the model proposed by [1] with those of the KAS, OFW, Friedman or Kissinger methods, which is thus an original feature. To conclude, while comparative investigations often rely on an analysis of pure biopolymers and/or a limited number of feedstocks, we selected not less than 5 different biomass types herein, namely spruce wood, wheat straw, swine manure, miscanthus and switchgrass, to represent forest biomass, by-products of Canadian agriculture, by-products of livestock, and energy crops, respectively. Each of these feedstocks was thermally treated using a thermogravimetric analyzer following the methodology described in Sect. “Experiments”. As for the modeling procedures, they are described in Sect. “Kinetic modeling”. The obtained results will then be analyzed in Sect. “Results and discussion” to determine the modeling approaches leading to the best agreement between measured and simulated conversion degree profiles. Although the emphasis is on kinetic modeling herein, and not on the intrinsic analysis of the pyrolysis behavior of each tested feedstock, thermodynamic parameters, namely enthalpy, Gibbs free energy, and entropy, will still be calculated based on the parameters issued from the use of the isoconversional methods before being discussed, for completeness. Finally, conclusions will be drawn to highlight the potential strengths and weaknesses of the assessed models. As such, this study has the potential to provide insight into how to select and parameterize particular kinetic modeling approaches depending on their advantages with respect to the targeted application.

## Experiments

### Feedstocks

Table 1 displays the proximate and ultimate analyses of the different feedstocks analyzed, namely spruce wood (SW), wheat straw (WS), swine manure (SM), miscanthus (M) and switchgrass (SG).

**Table 1:** Proximate and ultimate analyses of tested biomass samples

Sample	Proximate analysis/wmass%—dry basis (db)			Ultimate analysis/mass%—dry ash free basis (daf)				
	Volatiles	Fixed carbon*	Ash	Carbon	Hydrogen	Nitrogen	Sulfur	Oxygen
SW	85.6	13.9	0.5	48.3	6.0	0.0	0.0	45.7
WS	79.3	11.7	9.0	47.4	6.1	0.7	0.1	45.7
SM	68.8	21.5	9.7	45.2	5.7	3.9	1.0	44.2
M	83.3	14.4	2.3	47.5	6.1	1.0	0.1	45.3
SG	85.5	12.7	1.8	48.8	6.2	0.9	0.1	44.0

*Calculated by difference

Regarding the biochemical analysis and inorganic content of tested samples, they are provided in Table 2.

**Table 2:** Biochemical analysis and mineral matter content of tested biomass samples

Sample	Biochemical analysis/mass%—db			Inorganic content/ppm—db							
	Cellulose	Hemicellulose	Lignin	Al	Ca	Fe	K	Mg	Na	P	S
SW	54.5	11.8	26.5	20	898	29	686	161	5	27	44
WS	37.1	19.0	16.9	40	2997	47	32,928	1796	4773	585	1621
SM	12.4	12.7	6.7	225	24,235	8640	15,275	5727	3793	10,231	8118
M	51.1	25.8	12.3	100	3644	119	3126	882	101	688	672
SG	47.6	24.3	11.4	48	3369	82	1685	721	12	440	443

It is noteworthy that the biopolymer contents summarized in Table 2 are globally in line with previously reported biochemical analyses from the literature. Indeed, the cellulose, hemicellulose and lignin mass% of SW are relatively close to those estimated in [21, 22]. Similarly, the composition depicted in Table 2 for WS is consistent with those obtained in [23, 24]. As for SM, the cellulose, hemicellulose and lignin contents reported in [25–28] were comprised between 9.0 and 15.2 mass%, 14.2 and 24.9 mass% and 0.9 and 8.0 mass%, respectively, which, here again, globally matches the results depicted in Table 2. To conclude, the biochemical compositions obtained for M and SG are, on the whole, close to those issued from [29] and [30], respectively.

As far as sample preparation is concerned, each feedstock was ground and sieved into a size fraction less than 125 μm to limit the effect of temperature lags and gradients which can distort the kinetic parameters derived from pyrolysis experiments [31, 32]. Samples were then dried in an oven at 105 °C for 24 h before being analyzed.

### TGA analyses

A SETARAM SETSYS Evolution thermogravimetric analyzer (TGA) was used to perform the non-isothermal pyrolysis tests following a procedure similar to the one described in [13–15, 33]. A constant 60 mL min^−1^ nitrogen flow was continuously used to maintain an inert atmosphere around the samples during the measurements. Samples were first heated for 15 min at 378 K to eliminate free water. The temperature was then increased up to 1223 K using heating rates of 5, 10, 15 or 30 K min^−1^, with a plateau of 30 min at this temperature. Three tests were performed for each sample and operating condition as in [14, 15, 33] to check the reproducibility of obtained data. The mass loss profiles presented in the following and used for kinetic modeling purposes thus correspond to averaged ones, noting that uncertainties reported in Sect. “Kinetic modeling” were estimated based on a 95% confidence level (see [15, 33] for additional information regarding measurement uncertainties). As for the conversion degree $\alpha$ at any given time $t$ (expressed in s), it was calculated from the initial and final residual masses (${m}_{0}$ and ${m}_{\infty }$ assessed for temperatures of 379 and 1223 K, respectively) based on Eq. (1):

1 $$\alpha = 100\frac{{\left( {m_{0} - m_{{\text{t}}} } \right)}}{{\left( {m_{0} - m_{\infty } } \right)}}$$

## Kinetic modeling

### Model-free methods

The variation of the fuel conversion degree $\alpha$ as a function of time $t$ follows an Arrhenius equation of type:

2 $$\frac{{{\text{d}}\alpha }}{{{\text{d}}t}} = A\exp \left( { - \frac{{E_{{\text{a}}} }}{RT}} \right)f\left( \alpha \right)$$

where $A$ (s^−1^) is the pre-exponential factor, ${E}_{{\text{a}}}$ (J mol^−1^) denotes the activation energy, $R$ (8.314 J mol^−1^ K^−1^) is the ideal gas constant, $T$ (K) stands for the temperature, while $f\left(\alpha \right)$ represents the reaction model (see Table 3), noting that 17 reaction models, namely F1, F2, F3, F4, F5, D2, D3, R2, R3, A2, A3, A4, A5, P2, P3, P4 and P5, were tested in the present work.

**Table 3:** Summary of some commonly used reaction models

Reaction model	Denomination	$f\left(\alpha \right)$	$g(\alpha )$
Order-based	Mampel first-order (F1)	$(1-\alpha )$	$-{\text{ln}}(1-\alpha )$
	$n$-th order (F$n$)	${(1-\alpha )}^\textrm{n}$	$\frac{{\left(1-\alpha \right)}^{-\left(\textrm{n}-1\right)}-1}{n-1}$
Diffusion	2-D diffusion (D2)	${[-{\text{ln}}\left(1-\alpha \right)]}^{-1}$	$\left(1-\alpha \right){\text{ln}}\left(1-\alpha \right)+\alpha$
	3-D diffusion Jander (D3)	$\frac{3}{2}{(1-\alpha )}^{2/3}{[1-{\left(1-\alpha \right)}^\frac{1}{3}]}^{-1}$	${[1-{\left(1-\alpha \right)}^\frac{1}{3}]}^{2}$
Geometrical	Contracting cylinder (R2)	${2(1-\alpha )}^{1/2}$	$1-{\left(1-\alpha \right)}^{1/2}$
	Contracting sphere (R3)	${3(1-\alpha )}^{2/3}$	$1-{\left(1-\alpha \right)}^{1/3}$
Nucleation	Avrami–Erofeev (A$n$) (n ≥ 2)	$n(1-\alpha ){[-{\text{ln}}(1-\alpha )]}^\textrm{(n-1)/n}$	${[-{\text{ln}}\left(1-\alpha \right)]}^{1/\textrm{n}}$
Power law	$n$ Power law (P$n$)	$n{\alpha }^\textrm{(n-1)/n}$	${\alpha }^\textrm{1/n}$

Since the heating rate $\beta$ during non-isothermal analyses expresses as $\beta ={\text{d}}T/{\text{d}}t$, Eq. (2) can be rewritten so that the final form of the decomposition kinetics of the studied samples can be expressed as follows:

3 $$\frac{{{\text{d}}\alpha }}{{{\text{d}}T}} = \frac{A}{\beta }\exp \left( { - \frac{{E_{{\text{a}}} }}{RT}} \right)f\left( {\upalpha } \right)$$

#### Isoconversional methods

Isoconversional methods (also referred to as model-free approaches) consider that the activation energy only depends on the conversion degree, and not on the heating rate [9]. Among existing isoconversional methods, we considered the Kissinger–Akahira–Sunose (KAS), Ozawa–Flynn–Wall (OFW) and Friedman models, which are widely used in biomass pyrolysis studies, as exemplified in Sect. “Introduction”. Their governing equations are briefly summarized below.

First, and as for the KAS model, rearranging and integrating Eq. (3) based on the Coats–Redfern approximation [34] as detailed in [14, 15] leads to the following relation, in which the subscripts $\alpha$ and $i$ refer to given conversion degrees and heating rates, respectively, while $g\left(\alpha \right)$ represents the integral form of the reaction model (see Table 3):

4 $$\ln \left( {\frac{{\beta_{{\text{i}}} }}{{T_{{{\upalpha },{\text{i}}}}^{2} }}} \right) = \ln \left[ {\frac{{A_{{\upalpha }} R}}{{E_{{{\text{a}},{\upalpha }}} g\left( \alpha \right)}}} \right] - \frac{{E_{{{\text{a}},{\upalpha }}} }}{{RT_{{{\upalpha },{\text{i}}}} }}$$

Regarding the OFW model, its expression, which is based on the Doyle’s approximation [35] can be written as follows:

5 $$\ln \beta_{{\text{i}}} = \ln \left[ {\frac{{A_{{\upalpha }} E_{{{\text{a}},{\upalpha }}} }}{Rg\left( \alpha \right)}} \right] - 5.331 - 1.052\frac{{E_{{{\text{a}},{\upalpha }}} }}{{RT_{{{\upalpha },{\text{i}}}} }}$$

Finally, the equation related to the Friedman differential method [36] is given as per Eq. (6) [37]:

6 $$\ln \left[ {\beta_{{\text{i}}} \left( {\frac{{{\text{d}}\alpha }}{{{\text{d}}T}}} \right)_{{{\upalpha },{\text{i}}}} } \right] = \ln \left[ {A_{{\upalpha }} f\left( \alpha \right)} \right] - \frac{{E_{{{\text{a}},{\upalpha }}} }}{{RT_{{{\upalpha },{\text{i}}}} }}$$

By plotting the evolution of ${\text{ln}}\left(\frac{{\beta }_{{\text{i}}}}{{T}_{\mathrm{\alpha },{\text{i}}}^{2}}\right)$, ${\text{ln}}{\beta }_{{\text{i}}}$ and ${\text{ln}}\left[{\beta }_{{\text{i}}}{\left(\frac{{\text{d}}\alpha }{{\text{d}}T}\right)}_{\mathrm{\alpha },{\text{i}}}\right]$ as a function of $\frac{1}{{T}_{\mathrm{\alpha },{\text{i}}}}$ for different ${\beta }_{{\text{i}}}$ when considering the KAS, OFW and Friedman models, respectively, one obtains linearized straight lines whose slopes allow deriving ${E}_{{\text{a}},\mathrm{\alpha }}$ values for each conversion degree, while the intercept allows deriving the ${A}_{\mathrm{\alpha }}$ values once a proper reaction model is selected (see Sect. “Identification of proper reaction models in the case of model-free methods”).

#### Kissinger method

The 1-step Kissinger method [38] is based on the determination of the peak temperature ${T}_{{\text{max}}}$ corresponding to the maximum reaction rate to infer an overall activation energy ${E}_{{\text{a}}}$ based on Eq. (7), as detailed in [9].

7 $$\ln \frac{\beta }{{T_{{{\text{max}}}}^{2} }} = \ln \frac{AR}{{E_{{\text{a}}} }} - \frac{{E_{{\text{a}}} }}{{RT_{{{\text{max}}}} }}$$

By plotting the evolution of ${\text{ln}}\frac{\beta }{{T}_{{\text{max}}}^{2}}$ as a function of $\frac{1}{{T}_{{\text{max}}}}$ for different $\beta$ values, straights are obtained, with their slopes and intercepts allowing to infer the global activation energy value and the pre-exponential factor.

**Fig. 1:**
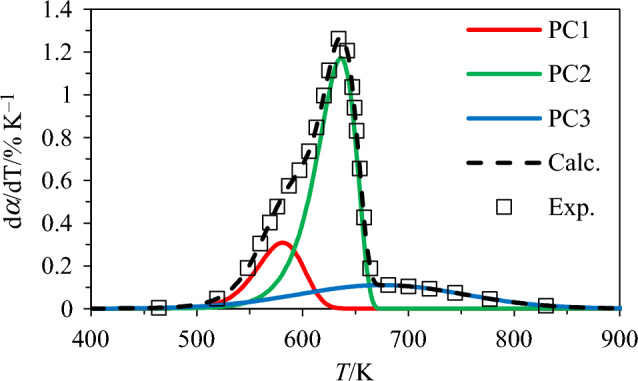
Deconvolution results obtained using the Fraser-Suzuki function for SW and $\beta$=10 K min^−1^ with PC1 = hemicellulose, PC2 = cellulose and PC3 = lignin

To better account for the successive decomposition of the different biopolymers or pseudo-components (PC) making up biomass (i.e., hemicellulose, cellulose and lignin), pyrolysis can be further assumed to follow three parallel and independent reactions [9, 39]. To that end, one has first to resolve the overlapped profiles of the conversion rate (${\text{d}}\alpha /{\text{d}}T$) into several independent curves. This can be achieved by using the Fraser-Suzuki (FS) deconvolution method, which treats the ${\text{d}}\alpha /{\text{d}}T$ signal of the $j$^th^ PC as a function of the temperature following [40, 41]:

8 $$\left( {\frac{{{\text{d}}\alpha }}{{{\text{d}}T}}} \right)_{{\text{j}}} = H_{{{\text{p}},{\text{j}}}} \exp \left[ { - \frac{\ln 2}{{A_{{{\text{s}},{\text{j}}}}^{2} }}\ln \left( {1 + 2A_{{{\text{s}},{\text{j}}}} \frac{{T - T_{{{\text{max}},{\text{j}}}} }}{{w_{{{\text{hf}},{\text{j}}}} }}} \right)^{2} } \right]$$

where ${H}_{{\text{p}}}$, ${w}_{{\text{hf}}}$, and ${A}_{{\text{s}}},$ respectively, stand for the maximum peak height (K^−1^), the half-width of the peak (K) and the asymmetry (dimensionless) of the ${\text{d}}\alpha /{\text{d}}T$ versus $T$ profile for the $j$^th^ PC. The values of these parameters (initially set as in [40]) were optimized for each feedstock (using the generalized reduced gradient solver previously implemented in [42]) so as to minimize an objective function defined as a least square sum between the FS deconvolution of the ${\text{d}}\alpha /{\text{d}}T$ vs. *T* profile given by Eq. (9) (denoted “Calc.” in the following) and the experimental one (denoted “Exp.”) (see [40]):

9 $$\frac{{{\text{d}}\alpha }}{{{\text{d}}T}} = \mathop \sum \limits_{{{\text{j}} = 1}}^{3} c_{{\text{j}}} H_{{{\text{p}},{\text{j}}}} \exp \left[ { - \frac{\ln 2}{{A_{{{\text{s}},{\text{j}}}}^{2} }}\ln \left( {1 + 2A_{{{\text{s}},{\text{j}}}} \frac{{T - T_{{{\text{max}},{\text{j}}}} }}{{w_{{{\text{hf}},{\text{j}}}} }}} \right)^{2} } \right]$$

where ${c}_{{\text{j}}}$ is the proportion of the $j$^th^ PC calculated from the biochemical analysis provided in Table 2 so that $\sum_{{\text{j}}=1}^{3}{c}_{{\text{j}}}=1$. Once the ${\text{d}}\alpha /{\text{d}}T$ versus $T$ profile of each PC is separated as exemplified in the case of SW in Fig. 1, the peak temperature values of each pseudo-component can be estimated and the kinetic parameters underlying their pyrolysis can be inferred using the above Kissinger equation (see Eq. 7). Finally, a theoretical conversion degree profile can be computed to represent the decomposition of all the biomass samples by merging the results of the simulations carried out with every biopolymer while considering their relative mass percent in the considered feedstocks.

### Fitting method

The fitting method proposed by [1] expresses the temperature dependence of the rate constant $k(T)$ by means of a modified Arrhenius equation of the form:

10 $$k\left( T \right) = A\left( {\frac{T}{{1{\text{ K}}}}} \right)^{{\text{q}}} \exp \left( { - \frac{{E_{{\text{a}}} }}{RT}} \right)$$

where $q$ is a temperature parameter of the modified Arrhenius function (MAF). Considering the above expression of the MAF, Eq. (3) then turns into:

11 $$\frac{{{\text{d}}\alpha }}{{{\text{d}}T}} = \frac{A}{\beta }\left( {\frac{T}{{1{\text{ K}}}}} \right)^{{\text{q}}} \exp \left( { - \frac{{E_{{\text{a}}} }}{RT}} \right)f\left( \alpha \right)$$

with the reaction mechanism being expressed based on the following Šesták–Berggren equation [17]:

12 $$f\left( \alpha \right) = \alpha^{{\text{m}}} \left( {1 - \alpha } \right)^{{\text{n}}} \left[ { - {\text{ln}}\left( {1 - \alpha } \right)} \right]^{{\text{p}}}$$

where $m$, $n$ and $p$ are empirically obtained exponent factors. Rearranging and integrating Eq. (12) leads to the following relation:

13 $$\mathop \smallint \limits_{{\alpha_{0} }}^{\alpha } \frac{{{\text{d}}\alpha }}{f\left( \alpha \right)} = \frac{A}{\beta }\mathop \smallint \limits_{{T_{0} }}^{T} \left( {\frac{T}{{1{\text{K}}}}} \right)^{{\text{q}}} \exp \left( { - \frac{{E_{{\text{a}}} }}{RT}} \right){\text{d}}T$$

noting that the method proposed by [1] consists in calculating the left- and right hand-side integrals (corresponding to the so-called conversion function and temperature integrals denoted ${I}^{\mathrm{\alpha }}$ and ${I}^{{\text{T}}}$) using the midpoint rule for 10,000 integration subintervals, so that:

14 $$I^{{\upalpha }} \left( {\alpha_{1} ,\alpha_{2} } \right) = \mathop \smallint \limits_{{\alpha_{1} }}^{{\alpha_{2} }} \frac{{{\text{d}}\alpha }}{f\left( \alpha \right)}{ } \approx h_{{\upalpha }} \mathop \sum \limits_{k = 1}^{10000} \frac{1}{{f\left( {\alpha_{{\text{k}}} } \right)}}$$

and

15 $$I^{{\text{T}}} \left( {T_{1} ,T_{2} } \right) = \mathop \smallint \limits_{{T_{1} }}^{{T_{2} }} \left( {\frac{T}{{1{\text{K}}}}} \right)^{{\text{q}}} \exp \left( { - \frac{{E_{{\text{a}}} }}{RT}} \right){\text{d}}T \approx h_{{\text{T}}} \mathop \sum \limits_{k = 1}^{10000} \left( {\frac{T}{{1{\text{K}}}}} \right)^{{\text{q}}} \exp \left( { - \frac{{E_{{\text{a}}} }}{{RT_{{\text{k}}} }}} \right)$$

where ${\alpha }_{{\text{k}}}={\alpha }_{1}+\left(k-0.5\right){h}_{\mathrm{\alpha }},$ $k=1, 2,\dots , 10000,$ ${h}_{\mathrm{\alpha }}=({\alpha }_{2}-{\alpha }_{1})/10000$ and ${T}_{{\text{k}}}={T}_{1}+\left(k-0.5\right){h}_{{\text{T}}},$ ${h}_{{\text{T}}}=({T}_{2}-{T}_{1})/10000$. It is then possible to calculate a set of “constants” ${A}_{\upbeta ,\mathrm{\varsigma }}$ of the pre-exponential factor $A$ for each range $[{\alpha }_{\mathrm{\varsigma }} - {\alpha }_{\mathrm{\varsigma }+1}]$ and $[{T}_{\mathrm{\varsigma }} - {T}_{\mathrm{\varsigma }+1}]$ $(\mathrm{with}\;\varsigma = 1, 2, . . . ,\mathrm{ N})$ of the initial data table $\{{T}_{\mathrm{\varsigma }}, {\alpha }_{\mathrm{\varsigma }}\}$ using Eq. (16):

16 $$A_{{\upbeta ,\mathrm{\varsigma }}} = I^{\upalpha } \left( {\alpha_{\mathrm{\varsigma }} ,\alpha_{{\mathrm{\varsigma } + 1}} } \right)/I^{{\text{T}}} \left( {T_{\mathrm{\varsigma }} ,T_{{\mathrm{\varsigma } + 1}} } \right), \quad \varsigma = 1, 2, \ldots , N$$

Considering that the $A/\beta$ ratio is constant, the optimal values of the kinetic triplet ($A,{E}_{{\text{a}}},(m, n, p)$) are assumed to be found when the set of calculated constants ${A}_{\upbeta ,\mathrm{\varsigma }}$ exhibits a minimum scatter. An optimization procedure (based on the use of a generalized reduced gradient solver) must therefore be implemented in order to define the values that the above-listed parameters must take so that the following variation coefficient $F$ becomes minimal:

17 $$F = \frac{1}{{\overline{{A_{{\upbeta }} }} }}\sqrt {\frac{{\mathop \sum \nolimits_{i} \left( {A_{{{\upbeta },{\mathrm{\varsigma }}}} - \overline{{A_{{\upbeta }} }} } \right)^{2} }}{N}}$$

with $\overline{{A }_{\upbeta }}$ being the average of the ${A}_{\upbeta ,\mathrm{\varsigma }}$ constants. Of note, the calculation procedure described in [1] is originally applied independently for each $\beta$ value, thus leading to infer one set of rate constant parameters per heating rate. Nevertheless, and in accordance with the ICTAC Kinetics Committee recommendations [43–45], computing rate constant parameters based on measurements taken with a single heating rate must be avoided, and only multiple temperature program methods should be used for kinetic computations. It was notably shown in [43] that model-fitting methods can be as reliable as isoconversional ones provided that the models are fitted simultaneously to multiple data sets obtained under different temperature programs. As a consequence, and instead of applying the exact same methodology as the one described in [1], we proposed an alternative resolution scheme which consists in operating a global optimization (as we did in [33, 42]) aimed at minimizing the above $F$ function while simultaneously considering the data issued from the experiments conducted with the 4 heating rates, to obtain a single set of kinetic triplets for each studied feedstock. To that end, we used the generalized reduced gradient solver previously implemented in [42], to which the reader is referred for more information.

### Network model

The CPD model, initially developed by Grant et al. [46–48] to simulate coal devolatilization, is based on the percolation theory. It uses a lattice model to account for the fuel chemical structure, which corresponds to a polymer-like network of fused aromatic clusters connected by non-aromatic chemical bridges. During heating, labile bridges become unstable and may undergo breakage following the following reaction sequence:

18 $$\pounds\mathop\to\limits ^{{k_{{\text{b}}} }} \pounds^{*} \left\{ {\begin{array}{*{20}c} {\mathop\to\limits ^{{k_{{\updelta }} }} 2\delta \mathop\to\limits ^{{k_{{\text{g}}} }} 2g_{1} } \\ \!\!\!{\mathop\to\limits ^{{k_{{\text{c}}} }} c + 2g_{2} }\\\end{array} } \right.$$

where $\pounds$ is a labile bridge, ${\pounds }^{*}$ represents a reactive bridge intermediate, $\delta$ is a side chain, $c$ stands for a char bridge, ${g}_{1}$ and ${g}_{2}$ denote light gases, while ${k}_{{\text{b}}}$, ${k}_{\updelta }$, ${k}_{{\text{g}}}$ and ${k}_{{\text{c}}}$ stand for the Arrhenius-type rate constants of the different reactions depicted in Eq. (18). The bridge breaking process thus begins with the decomposition of a labile bridge $\pounds$ to form an activated complex ${\pounds }^{*}$, which rapidly reacts to generate either a side chain $\delta$ or char $c$ and gas ${g}_{2}$. The so-formed side chain can eventually undergo a cracking process to be converted into light gases ${g}_{1}$. To estimate the quantity of gaseous species emitted, the CPD model requires setting the values of five input parameters, namely the number of attachments per cluster (also called the coordination number $(\sigma +1)$), the initial fraction of intact bridges between clusters $({p}_{0})$, the initial fraction of stable bridges $({c}_{0})$ and the average molecular weights of aromatic clusters $({M}_{{\text{cluster}}})$ and side chains $({M}_{\updelta })$. As mentioned in Sect. “Introduction”), the CPD model was then extended to apply to biomass [20]. To that end, rate coefficients and structural parameters were proposed in [49] for cellulose, hemicellulose and lignin, as detailed in Table 4. One calculation is thus done for each pseudo-component, considering similar operating conditions (i.e., identical thermal histories). The devolatilized fraction of the whole biomass can then be computed, as was the case with the 3-step Kissinger method, by merging the results of the three calculations based on the mass percent of each biopolymer composing the studied feedstock.

**Table 4:** Rate coefficients and fuel structural parameters proposed in [49] for use in the bio-CPD model

	Cellulose	Hemicellulose	Lignin
*Rate coefficients*			
*E*_b_/kcal mol^−1^—bridge scission activation energy	55.4	51.5	55.4
*A*_b_/s^−1^—bridge scission pre-exponential factor	2.0E + 16	1.2E + 20	7.0E + 16
$\sigma$_b_/kcal mol^−1^—bridge scission standard deviation	4.1	0.1	0.5
*E*_g_/kcal mol^−1^—gas release activation energy	61.2	38.2	69.0
*A*_g_/s^−1^—gas release pre-exponential factor	3.0E + 15	3.0E + 15	2.3E + 19
$\sigma$_g_/kcal mol^−1^—gas release standard deviation	8.1	5.0	2.6
${k}_{\updelta }/{k}_{{\text{c}}}$—composite rate constant	100	1.35	1.7
*E*_cross_/kcal mol^−1^—cross-linking activation energy	65.0	65.0	65.0
*A*_cross_/s^−1^—cross-linking pre-exponential factor	3.0E + 15	3.0E + 15	3.0E + 15
*Structural parameters*			
${M}_{{\text{cluster}}}$—average molecular weight per cluster	81	77.5	208
${M}_{\updelta }$—average molecular weight per side chain	22.7	21.5	39
${p}_{0}$—initial fraction of intact bridges between clusters	1.0	1.0	0.71
$\sigma +1$—coordination number	3.0	3.0	3.5
${c}_{0}$—initial fraction of stable bridges	0.0	0.0	0.0

The code proposed by Perry et al. [50, 51] was used to perform the calculations as was previously done in [33, 42, 52]. The structural parameters listed in Table 4 were used and the mass percentages of each biopolymer composing the studied biomass were taken from Table 2.

### Identification of proper reaction models in the case of model-free methods

Based on the activation energies inferred using the model-free methods described in Sect. “Model-free methods”, the evolution of the conversion degree of each tested fuel as a function of temperature was simulated using Eq. (19) while considering the 17 reaction models listed in Sect. “Model-free methods”.

19 $$\left( {\frac{{{\text{d}}\alpha }}{{{\text{d}}T}}} \right)_{{{\text{sim}}}} = \frac{A}{\beta }\exp \left( { - \frac{{E_{{{\text{a}},{\upalpha }}} }}{RT}} \right)f\left( \alpha \right)$$

To that end, ${N}_{{\text{p}}}$=1550 theoretical points were computed on a conversion degree range extending from 2.5 to 80%. Once this is done, the most suitable reaction model can be identified, the latter corresponding to the one whose use leads to the best agreement between simulated and measured conversion degree profiles as verified based on the relative deviation index described in Sect. “Comparison between simulated and measured conversion degree profiles”.

### Comparison between simulated and measured conversion degree profiles

The relative ability of each tested model to properly reproduce experimentally monitored results was assessed by computing the root-mean-square deviation $({\text{RMSD}}$) between simulated and measured conversion degree profiles using Eq. (20).

20 $${\text{RMSD}} = { }\sqrt {\frac{{\mathop \sum \nolimits_{{T_{{{\text{exp}}.,{\text{l}}}} = T_{{2.5{\text{\% }}}} }}^{{T_{{{\text{exp}}.,{\text{l}}}} = T_{{80{\text{\% }}}} }} \left( {\alpha_{{{\text{exp}}.,{\text{T}}_{{{\text{exp}}.,{\text{l}}}} }} - \alpha_{{{\text{calc}}.,{\text{T}}_{{{\text{exp}}.,{\text{l}}}} }} } \right)^{2} }}{{N_{{\text{p}}} }}}$$

where the subscript $l$ denotes the $l$^th^ point of the series, while exp. and calc. refer to experimental and calculated conversion degrees, respectively. Finally, ${T}_{2.5\mathrm{\%}}$ and ${T}_{80\mathrm{\%}}$ represent the temperatures corresponding to $\alpha$ values of 2.5% and 80%.

### Thermodynamic parameters

In order to further characterize and analyze the pyrolysis behavior of the tested biomass, thermodynamic parameters, namely the changes of enthalpy ($\Delta H$), Gibbs free energy ($\Delta G$) and entropy ($\Delta S$) were calculated using the activation energy values inferred by means of the isoconversional models. To that end, the fundamental equation of the theory of the active complex were used as in [12, 53–56]. The change of enthalpy was thus calculated following Eq. (21), and was then used to obtain the change of Gibbs free energy based on Eq. (22), which reflects the favorability of reactions with respect to both the first and the second laws of thermodynamics.

21 $$\Delta H = E_{{{\text{a}},{\upalpha }}} - RT_{{{\text{max}}}} { }$$

22 $$\Delta G = E_{{{\text{a}},{\upalpha }}} + RT_{{{\text{max}}}} \ln \left( {\frac{{k_{{\text{B}}} T_{{{\text{max}}}} }}{{hA_{{\upalpha }} }}} \right){ }$$

with ${k}_{{\text{B}}}$ (1.381 × 10^–23^ J K^−1^) being the Boltzmann constant, $h$ (6.626 × 10^–34^ J s) the Plank constant, ${T}_{{\text{max}}}$ the derivate mass loss rate (dTG) peak temperature, while ${A}_{\mathrm{\alpha }}$ expresses as [53, 56]:

23 $$A_{{\upalpha }} = \beta_{{\text{i}}} E_{{{\text{a}},{\upalpha }}} \frac{{{\text{exp}}\left( {\frac{{E_{{{\text{a}},{\upalpha }}} }}{{RT_{{{\text{max}}}} }}} \right)}}{{RT_{{{\text{max}}}}^{2} }}{ }$$

Finally, the entropy change reflects how near the system is to its own thermodynamic equilibrium. This parameter (calculated based on Eq. (24)) notably allows distinguishing between the reactions which are considered as fast ($\Delta S$ > 0), slow ($\Delta S$ < 0) or normal ($\Delta S$ = 0). Actually, negative entropy changes indicate that the products formed are characterized by a higher degree of arrangement than the initial reactants, while the contrary suggest that the degree of randomness of preliminary reactants is greater than that of the products generated from the thermal degradation:

24 $$\Delta S = \frac{\Delta H - \Delta G}{{T_{{{\text{max}}}} }}{ }$$

## Results and discussion

### TGA results

**Fig. 2:**
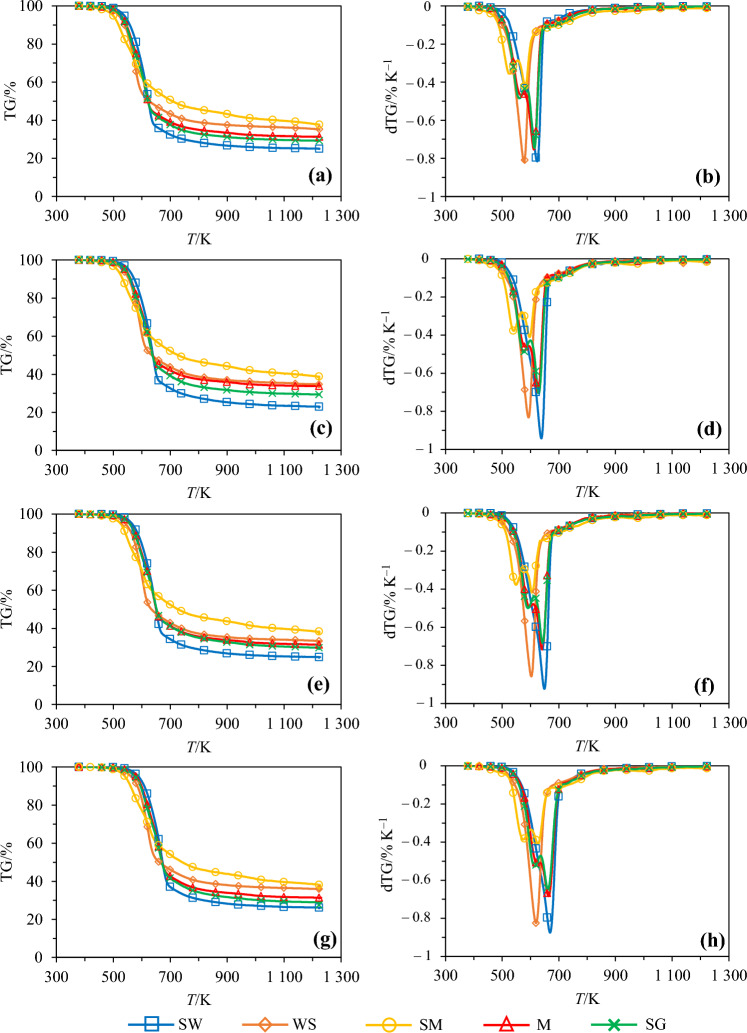
Evolution of mass loss noted ‘TG’ (**a**, **c**, **e** and **g**) and derivate mass loss rate noted ‘dTG’ (**b**, **d**, **f** and **h**) as a function of the temperature for spruce wood (SW), wheat straw (WS), swine manure (SM), miscanthus (M) and switchgrass (SG) and heating rates of 5 K min^−1^ (**a** and **b**), 10 K min^−1^ (**c** and **d**), 15 K min^−1^ (**e** and **f**) and 30 K min^−1^ (**g** and **h**)

Results issued from the TGA analyses conducted with each biomass are detailed in Fig. 2. Curves depicting variations of the mass loss (TG) and mass loss rate (dTG) as a function of the temperature are reported therein for heating rates of 5, 10, 15 and 30 K min^−1^. As can be seen by looking at the TG curves, the pyrolysis of each feedstock follows a three-stage process, which is consistent with the general trend reported in TGA-based analyses of biomass thermal degradation (see [22, 57–62], among others). An initial mass decrease is thus observed below ⁓ 450 K and corresponds to dehydration and removal of extractives. A rapid mass loss stage, in which the volatile components are removed due to the decomposition of biomass, is then observed for temperatures going up to 650–700 K, depending on the heating rate. Between ⁓ 60 and ⁓ 86% of the total mass loss is recorded during this second stage, which can be related to the rapid decomposition of hemicellulose and cellulose. It is noteworthy that contrary to the dTG curves obtained for WS and SW, which exhibit a single peak for mean temperatures of ⁓ 600 and ⁓ 650 K, as also observed in [21] for instance, the peaks of the dTG curves related to the other biomass samples are observed at ⁓ 605 K for SM and ⁓ 640 K for M and SG, with a shoulder located on the left, for a temperature ⁓40 K lower (see Fig. 2b, d, f and h), as also reported in [27, 62] and [23] for SM, M and SG, respectively. Such overlapping peaks actually produce a single dTG peak with a lower temperature shoulder, which represents the decomposition of hemicellulose, and a higher temperature peak, which accounts for the decomposition of cellulose. Of note, the shoulder peak in the case of SM, whose composition is very different from that of the other biomass samples analyzed herein, can be attributed, not only to the degradation of hemicellulose, but also to the decomposition of protein and lipids, as highlighted in [63]. Above 650–700 K, a tailing corresponding to the slow decomposition of lignin can be observed. This latter occurs over a broad range of temperatures, thus providing a gently sloping baseline to the dTG curves [64, 65].

Notwithstanding some of the above-described similarities (especially as far as the existence of a three-stage thermal degradation process for all tested biomass is concerned), the characteristic pyrolysis temperatures, mass loss rates and residual masses still differ from one feedstock to another. To better figure out these discrepancies, Table 5 reports the temperatures estimated for conversion degrees of 10, 50 and 90% (referred to as *T*_10%_, *T*_50%_, *T*_90%_), the temperature (denoted *T*_max_) for which the dTG peaks are recorded, in addition to the maximum mass loss rate (*dTG*_max_) and the residual mass at 1223 K (*TG*_1223 K_) measured with each feedstock.

**Table 5:** Characteristic decomposition temperatures (*T*_10%_, *T*_50%_, *T*_90%_ and *T*_max_), maximum mass loss rate (dTG_max_) and residual mass at 1223 K (TG_1223 K_) for the five investigated biomass and heating rates of 5, 10, 15 and 30 K min^−1^

	*T*_10%_ /K	*T*_50%_ /K	*T*_90%_ /K	*T*_max_ /K	dTG_max_ /% K^−1^	*TG*_1223 K_ /%	*T*_10%_ /K	*T*_50%_ /K	*T*_90%_ /K	*T*_max_ /K	dTG_max_ /% K^−1^	*TG*_1223 K_ /%
	*5 K min*^**−1**^						*10 K min*^**−1**^					
SW	549.5 *(*± *0.04)*	609.5 *(*± *0.37)*	700.7 *(*± *1.29)*	622.8 *(*± *0.26)*	− 0.88 *(*± *0.13)*	24.9 *(*± *3.43)*	566.3 *(*± *0.89)*	626.9 *(*± *0.90)*	730.0 *(*± *5.18)*	639.4 *(*± *0.97)*	− 0.96 *(*± *0.02)*	22.5 *(*± *0.62)*
WS	525.7 *(*± *0.03)*	576.7 *(*± *0.20)*	728 *(*± *0.99)*	577.4 *(*± *0.86)*	− 0.82 *(*± *0.02)*	34.9 *(*± *0.11)*	540.0 *(*± *2.46)*	591.0 *(*± *2.98)*	744.3 *(*± *2.99)*	591.9 *(*± *3.50)*	− 0.83 *(*± *0.06)*	34.2 *(*± *5.13)*
SM	505.5 *(*± *0.37)*	582.2 *(*± *0.34)*	904.2 *(*± *3.15)*	582.9 *(*± *1.26)*	− 0.43 *(*± *0.04)*	39.7 *(*± *1.21)*	520.1 *(*± *0.83)*	596.4 *(*± *0.80)*	919.8 *(*± *0.02)*	596.9 *(*± *1.11)*	− 0.42 *(*± *0.01)*	37.6 *(*± *0.05)*
M	535.2 *(*± *0.08)*	598.1 *(*± *0.16)*	713.6 *(*± *1.31)*	612 *(*± *1.07)*	− 0.75 *(*± *0.01)*	31.2 *(*± *0.48)*	549.6 *(*± *0.31)*	611.9 *(*± *0.06)*	724.2 *(*± *4.35)*	626.5 *(*± *0.19)*	− 0.65 *(*± *0.10)*	33.6 *(*± *4.34)*
SG	534.6 *(*± *0.62)*	599.7 *(*± *1.06)*	718.0 *(*± *0.85)*	613.8 *(*± *0.49)*	− 0.75 *(*± *0.08)*	29.1 *(*± *4.02)*	549.6 *(*± *0.24)*	614.3 *(*± *0.36)*	734.0 *(*± *4.39)*	629.2 *(*± *0.23)*	− 0.68 *(*± *0.13)*	29.3 *(*± *3.66)*
	*15 K min*^**−1**^						*30 K min*^**−1**^					
SW	575.6 *(*± *2.17)*	635.7 *(*± *1.45)*	730.1 *(*± *6.92)*	648.4 *(*± *1.22)*	− 0.89 *(*± *0.07)*	24.6 *(*± *4.35)*	598.3 *(*± *2.76)*	657.1 *(*± *2.22)*	743.4 *(*± *1.06)*	667.9 *(*± *2.28)*	− 0.88 *(*± *0.01)*	25.9 *(*± *1.83)*
WS	551.8 *(*± *1.27)*	602.1 *(*± *0.65)*	748.8 *(*± *2.92)*	602.9 *(*± *0.12)*	− 0.86 *(*± *0.01)*	32.8 *(*± *0.49)*	572.1 *(*± *2.06)*	621.9 *(*± *2.04)*	765.7 *(*± *3.46)*	620.5 *(*± *2.10)*	− 0.82 *(*± *0.03)*	35.0 *(*± *0.12)*
SM	531.8 *(*± *0.66)*	605.3 *(*± *0.43)*	934.3 *(*± *5.33)*	605.6 *(*± *0.32)*	− 0.42 *(*± *0.01)*	36.9 *(*± *0.01)*	547.7 *(*± *0.11)*	627.6 *(*± *0.92)*	962.1 *(*± *4.58)*	626.7 *(*± *0.28)*	− 0.42 *(*± *0.01)*	36.1 *(*± *0.77)*
M	564.9 *(*± *2.12)*	626.9 *(*± *1.41)*	742.4 *(*± *1.97)*	640.8 *(*± *0.77)*	− 0.72 *(*± *0.04)*	31.1 *(*± *1.02)*	589.3 *(*± *2.99)*	649.1 *(*± *3.75)*	758.6 *(*± *3.42)*	661.8 *(*± *3.91)*	− 0.68 *(*± *0.02)*	31.0 *(*± *0.19)*
SG	563.0 *(*± *1.32)*	626.9 *(*± *0.27)*	756.3 *(*± *11.9)*	641.2 *(*± *1.32)*	− 0.65 *(*± *0.03)*	29.1 *(*± *3.27)*	586.7 *(*± *0.35)*	649.1 *(*± *0.18)*	788.2 *(*± *1.91)*	661.7 *(*± *0.15)*	− 0.65 *(*± *0.01)*	27.5 *(*± *0.14)*

Note that the bracketed italic values represent uncertainties (see Sect. “TGA analyses”)

As can be seen, *T*_max_ are significantly higher for SW than for WS (from 622.8 to 667.9 K for SW versus 577.4 to 620.5 K for WS with $\beta$ values going from 5 to 30 K min^−1^). This observation is actually consistent with the higher cellulose content of the former (around 54.5 mass% for SW versus 37.1 mass% for WS) and with the higher mass% of hemicellulose in WS (19.0 mass%) as compared to SW (11.8 mass%). On the other hand, the fact that relatively close ${T}_{{\text{max}}}$ are observed for SW, M and SG can be related to their relatively similar cellulose contents (54.5, 51.1 and 47.6 mass%, respectively). Besides, it is noteworthy that the *T*_10%_ values reported in Table 5 are lower for WS than for SW (from 525.7 to 572.1 K vs. 549.5 to 598.3 K for $\beta$ comprised between 5 and 30 K min^−1^). This trend, which can be partly explained by the higher hemicellulose content of WS, may also originate from the higher calcium, potassium, magnesium and sodium contents of WS (Table 2), noting that such alkali and alkaline earth metals (AAEMs) are known to shift the pyrolysis reactions to lower temperatures [4, 13, 14]. Similarly, SM which shows the lowest *T*_10%_ values (between 505.5 and 547.7 K for $\beta$ between 5 and 30 K min^−1^) is also the feedstock which presents the highest contents of inorganic species (especially as far as magnesium and calcium are concerned). The higher *TG*_1223 K_ values measured with WS and SM are, moreover, consistent with the effect of AAEMs, which tend to enhance char formation [4, 13], although the complexity of SM makes it rather difficult to trace the higher residual masses measured with this feedstock solely to the influence of inorganic species. To conclude, while M and SG show very similar pyrolysis behavior, with identical characteristic temperature, dTG_max_ and TG_1223 K_ values, much lower dTG_max_ are alternatively measured with SM, which has the lowest biopolymer content (see Table 2).

### Estimation of activation energy values

#### Isoconversional models

**Fig. 3:**
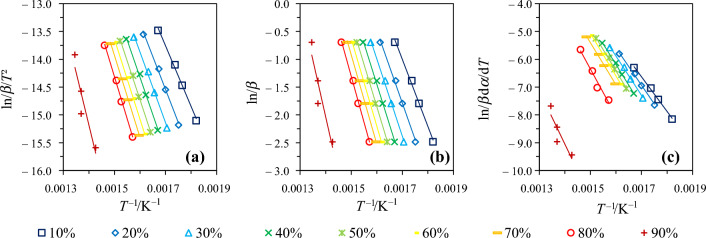
Modeling results issued from the use of the **a** KAS, **b** OFW and **c** Friedman models in the case of SW as an example

Following the methodology described in Sect. “Isoconversional methods”, we plotted a series of curves depicting the evolution of ${\text{ln}}\left(\frac{{\beta }_{{\text{i}}}}{{T}_{\mathrm{\alpha },{\text{i}}}^{2}}\right)$, ${\text{ln}}{\beta }_{{\text{i}}}$ and ${\text{ln}}\left[{\beta }_{{\text{i}}}{\left(\frac{{\text{d}}\alpha }{{\text{d}}T}\right)}_{\mathrm{\alpha },{\text{i}}}\right]$ as a function of $\frac{1}{{T}_{\mathrm{\alpha },{\text{i}}}}$ for the KAS, OFW and Friedman models, respectively, in order to infer ${E}_{{\text{a}},\mathrm{\alpha }}$ values based on the slopes of the so-obtained curves. Typical curves obtained in the case of SW for $\alpha$ values comprised between 10 and 90% are proposed in Fig. 3 as an example (results obtained with other biomass samples are not reported for brevity).

**Fig. 4:**
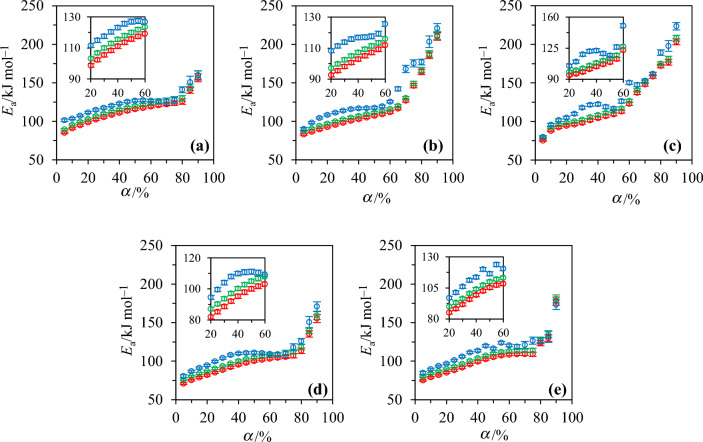
Evolution of the activation energies estimated by means of the KAS (red markers), OFW (green markers) and Friedman (blue markers) models as a function of the conversion degree for **a** SW, **b** WS, **c** SM, **d** M and **e** SG. The insets correspond to a zoom-in on the conversion degree domain extending from 20 to 60%. Note that the legends of the x- and y-axis labels of the main figures also apply to the insets

As mentioned in Sect. “TGA analyses”, results from multiple tests were averaged to mitigate the slight deviations potentially observed from test to test due to measurement noise. Doing so enabled obtaining good linear correlations, as exemplified by the plots of Fig. 3, as well as by the high determination coefficients (*R*^2^) reported in Table 6, which gathers the activation energies estimated based on the KAS, OFW and Friedman models. As can be seen by looking at the results from Table 6, the ${E}_{{\text{a}},\mathrm{\alpha }}$ values inferred using the KAS and OFW models globally merge on a single curve with a mean relative deviation of the order of 4%. Alternatively, the Friedman method leads to ${E}_{{\text{a}},\mathrm{\alpha }}$ values which are between 0.7 and 30.6% higher than the ${E}_{{\text{a}},\mathrm{\alpha }}$ obtained when averaging the activation energies issued from the KAS and OFW models, depending on the considered biomass and conversion degree. On the whole, the mean relative deviation between the ${E}_{{\text{a}},\mathrm{\alpha }}$ estimated using the Friedman and KAS models is 11.6% versus 7.4% when comparing the Friedman and OFW methods. Despite these discrepancies, all tested models predict a continuous increase of the activation energy with the conversion degree, as illustrated in Fig. 4, which is consistent with expectations since the species emitted at high temperatures typically require more energy to be released. The plots from Fig. 4 also show that the ${E}_{{\text{a}},\mathrm{\alpha }}$ values increase slightly for $\alpha$<80%, regardless of the considered model, while they rise much more significantly for $\alpha$>80%. Such an increase of the activation energies for high conversion degrees may be related to the decomposition of lignin, which contains a more rigid carbon–carbon linkage whose breakage thus requires more energy [66]. To conclude, it is noteworthy that the mean activation energy obtained for 20% < $\alpha$<80% with the Friedman model in the case of SW is 124.67 kJ mol^−1^, which is similar to the 119.08 kJ mol^−1^ found by [22] using the same feedstock and the same modeling approach. As for WS, the ${E}_{{\text{a}},\mathrm{\alpha }}$ found herein with the KAS and OFW models for conversion degrees between 10 and 80% (⁓90 and ⁓165 kJ mol^−1^ on average) are close to those estimated by [67], using the same isoconversional methods (⁓110 and ⁓183 kJ mol^−1^) while being higher than the mean activation energies estimated in [68] with the KAS (63.4 kJ mol^−1^) and OFW (65.2 kJ mol^−1^) methods.

**Table 6:** Activation energies assessed using the KAS, OFW and Friedman models. Note that the bracketed italic values represent uncertainties (see Sect. “TGA analyses”)

*α*/%	SW		WS		SM		M		SG	
	*E*_a,α_/kJ mol^−1^	*R*^2^	*E*_a,α_/kJ mol^−1^	*R*^2^	*E*_a,α_/kJ mol^−1^	*R*^2^	*E*_a,α_/kJ mol^−1^	*R*^2^	*E*_a,α_/kJ mol^−1^	*R*^2^
*KAS*										
10	91.0 *(*± *1.4)*	0.995	86.3 *(*± *1.1)*	0.994	87.7 *(*± *0.3)*	0.997	75.2 *(*± *1.1)*	0.984	79.0 *(*± *0.5)*	0.989
20	98.7 *(*± *1.3)*	0.995	92.6 *(*± *1.2)*	0.994	94.4 *(*± *0.3)*	0.995	81.9 *(*± *1.4)*	0.985	85.1 *(*± *0.3)*	0.988
30	105.5 *(*± *1.4)*	0.996	98.2 *(*± *1.3)*	0.994	98.1 *(*± *0.9)*	0.982	88.6 *(*± *1.6)*	0.985	91.6 *(*± *0.3)*	0.989
40	111.1 *(*± *1.4)*	0.996	102.9 *(*± *1.4)*	0.995	104.2 *(*± *1.0)*	0.982	95.2 *(*± *1.8)*	0.985	99.2 *(*± *0.5)*	0.991
50	115.7 *(*± *1.5)*	0.997	107.2 *(*± *1.5)*	0.995	109.2 *(*± *0.7)*	0.987	100.0 *(*± *2.0)*	0.986	105.3 *(*± *0.6)*	0.991
60	119.2 *(*± *1.5)*	0.997	111.9 *(*± *1.5)*	0.995	122.9 *(*± *0.9)*	0.988	103.1 *(*± *2.0)*	0.987	108.4 *(*± *0.6)*	0.992
70	122.2 *(*± *1.7)*	0.997	126.9 *(*± *1.9)*	0.994	147.9 *(*± *0.9)*	0.995	105.4 *(*± *2.0)*	0.987	109.1 *(*± *0.6)*	0.992
80	125.8 *(*± *1.7)*	0.996	164.2 *(*± *3.6)*	0.998	171.8 *(*± *2.7)*	0.990	113.3 *(*± *2.2)*	0.978	123.5 *(*± *3.5)*	0.987
90	156.5 *(*± *3.6)*	0.866	210.4 *(*± *5.2)*	0.988	203.3 *(*± *5.1)*	0.981	155.1 *(*± *3.9)*	0.964	178.0 *(*± *4.5)*	0.970
*OFW*										
10	95.6 *(*± *1.4)*	0.996	90.7 *(*± *1.1)*	0.995	91.7 *(*± *0.3)*	0.998	80.4 *(*± *1.1)*	0.988	84.0 *(*± *0.5)*	0.992
20	103.2 *(*± *1.3)*	0.996	97.1 *(*± *1.2)*	0.995	98.3 *(*± *0.3)*	0.996	87.0 *(*± *1.4)*	0.988	90.1 *(*± *0.3)*	0.991
30	109.9 *(*± *1.4)*	0.996	102.5 *(*± *1.3)*	0.996	102.2 *(*± *0.9)*	0.985	93.6 *(*± *1.6)*	0.988	96.5 *(*± *0.3)*	0.991
40	115.5 *(*± *1.4)*	0.997	107.2 *(*± *1.4)*	0.996	108.3 *(*± *1.0)*	0.985	100.2 *(*± *1.8)*	0.988	103.9 *(*± *0.5)*	0.993
50	120.0 *(*± *1.5)*	0.997	111.4 *(*± *1.5)*	0.996	113.4 *(*± *0.7)*	0.990	104.9 *(*± *1.9)*	0.988	109.9 *(*± *0.6)*	0.993
60	123.4 *(*± *1.5)*	0.998	116.0 *(*± *1.5)*	0.996	126.7 *(*± *0.9)*	0.990	108.0 *(*± *2.0)*	0.989	113.1 *(*± *0.6)*	0.994
70	126.4 *(*± *1.6)*	0.998	130.4 *(*± *1.9)*	0.995	151.2 *(*± *0.9)*	0.995	110.4 *(*± *2.0)*	0.989	113.9 *(*± *0.6)*	0.994
80	130.0 *(*± *1.7)*	0.997	166.6 *(*± *3.6)*	0.998	175.0 *(*± *2.7)*	0.991	118.1 *(*± *2.2)*	0.982	127.8 *(*± *3.5)*	0.990
90	160.1 *(*± *3.6)*	0.882	211.8 *(*± *5.2)*	0.989	208.0 *(*± *5.1)*	0.984	159.1 *(*± *3.9)*	0.969	180.7 *(*± *4.5)*	0.976
*Friedman*										
10	103.6 *(*± *1.8)*	0.997	98.3 *(*± *1.3)*	0.998	95.6 *(*± *1.4)*	0.994	87.0 *(*± *1.8)*	0.990	89.6 *(*± *1.8)*	0.990
20	111.5 *(*± *1.8)*	0.996	108.3 *(*± *1.3)*	0.998	104.9 *(*± *1.3)*	0.991	94.6 *(*± *1.9)*	0.990	96.8 *(*± *1.2)*	0.992
30	117.5 *(*± *1.9)*	0.997	113.9 *(*± *1.4)*	0.997	118.0 *(*± *1.3)*	0.989	104.0 *(*± *1.9)*	0.989	106.0 *(*± *0.6)*	0.994
40	123.1 *(*± *1.9)*	0.997	116.8 *(*± *1.5)*	0.997	122.2 *(*± *0.9)*	0.994	109.9 *(*± *2.1)*	0.989	113.6 *(*± *0.8)*	0.996
50	126.9 *(*± *2.1)*	0.998	117.7 *(*± *1.7)*	0.995	116.6 *(*± *1.2)*	0.988	111.0 *(*± *2.4)*	0.990	116.4 *(*± *1.0)*	0.996
60	126.9 *(*± *2.2)*	0.997	125.6 *(*± *1.6)*	0.992	150.3 *(*± *2.1)*	0.993	109.2 *(*± *2.4)*	0.989	120.5 *(*± *2.2)*	0.993
70	125.7 *(*± *2.3)*	0.996	168.0 *(*± *4.7)*	0.999	152.8 *(*± *1.8)*	0.998	109.5 *(*± *2.3)*	0.986	121.1 *(*± *2.7)*	0.989
80	141.1 *(*± *2.4)*	0.966	177.0 *(*± *3.8)*	0.999	189.3 *(*± *3.0)*	0.979	125.7 *(*± *3.5)*	0.950	126.5 *(*± *6.3)*	0.985
90	159.6 *(*± *5.6)*	0.786	220.8 *(*± *6.1)*	0.973	223.5 *(*± *4.9)*	0.993	170.8 *(*± *4.7)*	0.961	173.4 *(*± *8.1)*	0.937

Regarding M, [69] reported activation energies comprised between 113 and 143 kJ mol^−1^, while [70] computed ${E}_{{\text{a}},\mathrm{\alpha }}$ values for SG varying in the 109–134 kJ mol^−1^ range for $\alpha$ < 80% when implementing the KAS method, which, here again, is globally consistent with the order of magnitude of the ${E}_{{\text{a}},\mathrm{\alpha }}$ values reported in Table 6 (of further note, the feedstocks tested herein exhibit compositions (especially in terms of biopolymer contents) which are quite different from those of the biomass analyzed in [69, 70]). Finally, the results issued from the implementation of the KAS and OFW models in the case of SM for $\alpha$ < 60% (between ⁓ 90 and ⁓ 125 kJ mol^−1^ on average) are well in line with those reported by [27] (between ⁓85 and ⁓145 kJ mol^−1^). Furthermore, the ${E}_{{\text{a}},\mathrm{\alpha }}$ issued from Table 6 for SM also match very well the data from [63], who notably found a mean activation energy close to 130 kJ mol^−1^ for $\alpha$ < 80% when using the Friedman model, versus around 128 kJ mol^−1^ herein. Although the main purpose of this benchmarking analysis was not necessarily to infer ${E}_{{\text{a}}}$ values (the focus being indeed more specifically on the assessment of the relative ability of the different modeling approaches tested to properly reproduce measured data, as explained in Sect. “Introduction” and detailed in Sect. “Simulation of conversion degree profiles”, the above comparison betweenthe activation energies we inferred and those previously reported in the literature was still highly valuable as it allowed to validate the consistency of the results we obtained, while corroborating the overall relevance of the experimental and numerical methodologies described in Sects. “Experiments” and “Kinetic modeling”.

#### Kissinger and model-fitting approaches

The activation energies estimated when processing the data issued from the analysis of the 5 kinds of biomass while using the 1-step and 3-step Kissinger methods as well as the advanced fitting method from [1] are summarized in Table 7.

**Table 7:** Activation energies assessed using the 1-step and 3-step Kissinger methods together with the model-fitting approach from [1]

SW		WS		SM		M		SG	
*E*_a,α_/kJ mol^−1^	*R*^2^	*E*_a,α_/kJ mol^−1^	*R*^2^	*E*_a,α_/kJ mol^−1^	*R*^2^	*E*_a,α_/kJ mol^−1^	*R*^2^	*E*_a,α_/kJ mol^−1^	*R*^2^
*1-step Kissinger*									
127.3 *(*± *1.7)*	0.999	113.0 *(*± *1.7)*	0.998	114.0 *(*± *0.9)*	0.999	108.0 *(*± *2.3)*	0.991	114.6 *(*± *0.3)*	0.996
*3-step Kissinger*^†^									
100.1 *(*± *1.8)*	0.991	92.2 *(*± *0.7)*	0.991	84.5 *(*± *1.1)*	0.985	80.8 *(*± *1.5)*	0.989	84.5 *(*± *1.3)*	0.984
119.2 *(*± *1.7)*	0.996	111.2 *(*± *1.3)*	0.989	109.5 *(*± *2.1)*	0.992	103.6 *(*± *2.0)*	0.996	110.5 *(*± *1.3)*	0.991
149.0 *(*± *2.4)*	0.981	138.0 *(*± *5.4)*	1.000	120.2 *(*± *7.9)*	0.982	146.9 *(*± *3.3)*	1.000	142.3 *(*± *2.5)*	0.990
*Model-fitting approach from *[1]									
86.0 *(*± *0.1)*	–	107.9 *(*± *0.0)*	–	96.9 *(*± *1.1)*	–	85.3 *(*± *0.3)*	–	87.1 *(*± *0.2)*	–

Note that the bracketed italic values represent uncertainties (see Sect. “TGA analyses”)

^†^The first, second and third steps (corresponding to the first, second and third lines of the ‘3-step Kissinger’ section of the table) relate to PC1 (hemicellulose), PC2 (cellulose) and PC3 (lignin), respectively

As can be seen, the ${E}_{{\text{a}}}$ assessed by means of the 1-step Kissinger model are consistent with the mean values estimated in Sect. “Isoconversional models” based on the KAS and OFW methods. The relative deviation between the ${E}_{{\text{a}}}$ values from Table 7 and the mean ${E}_{{\text{a}},\mathrm{\alpha }}$ calculated based on the KAS and OFW models on the entire range of conversion degrees considered is indeed 6.7% on average, with values going from 0.5% for M to 14.2% for SM. As far as the 3-step Kissinger method is concerned, it predicts activation energies in increasing order as follows: pseudo-hemicellulose (PC 1) < pseudo-cellulose (PC 2) < pseudo-lignin (PC 3) for all the tested feedstocks, which is consistent with the conclusions from different studies using the Fraser-Suzuki deconvolution method to subsequently assess kinetic triplets of PC in biomass-containing samples by means of isoconversional approaches (see [40, 41] for instance). It is, however, noteworthy that the activation energies proposed in the literature for the 3 pseudo-components significantly vary from one study to another, thus making a direct comparison and/or validation of the results obtained herein not straightforward. As an example, based on the FS deconvolution parameters from [40, 71], one can compute activation energy values going from 71 [71] to 491 kJ mol^−1^ [40] for lignin (PC3) when using the Kissinger equation (see Sect. “Kissinger method”). That being said, it is of interest to mention that such a range of values encompasses the activation energies reported in Table 7 for this specific biopolymer. Although the values we found for PC1 and PC2 can be regarded as somewhat low, they are still globally in line with those estimated by [9, 72] (between 95.39 and 108.65 kJ mol^−1^ using combined kinetic and Kissinger methods) and [73] (⁓ 114 kJ mol^−1^ on average for 5% < $\alpha$ < 95% using the OFW model) when studying the pyrolysis of pure hemicellulose and cellulose, respectively. To conclude, the implementation of the fitting method proposed by [1] leads to lower estimates of the global activation energy for each biomass as compared to the values assessed using the 1-step Kissinger model. ${E}_{{\text{a}}}$ that are 19.4% lower on average are indeed obtained with relative deviations comprised between 4.5% for WS and 32.4% for SW.

### Simulation of conversion degree profiles

#### Identification of suitable reaction models

**Fig. 5:**
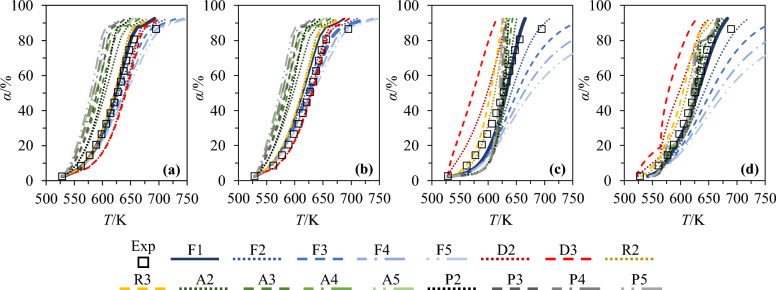
Comparison of conversion degree profiles measured with SW for $\beta$ = 10 K min^-1^ with simulated ones obtained from the implementation of the (**a**) KAS, (**b**) OFW, (**c**) 1-step Kissinger and (**d**) 3-step Kissinger methods with 17 different reaction models. Note that the results obtained with the best suited reaction models are plotted in each subfigure using thicker lines to ease their identification

In addition to the activation energies inferred in Sect. “Estimation of activation energy values”, the values of the pre-exponential factor integrated within the expression of the rate constant also need to be assessed to simulate experimental conversion degree profiles by means of model-free methods. To that end, the 17 reaction models listed in Sect. “Model-free methods” were tested with a view to identifying those leading to the best agreement between simulated conversion degree profiles and their experimental counterparts. Figure 5 depicts a series of profiles computed in the case of SW for a $\beta$ of 10 K min^−1^ as an example (results issued from the use of other $\beta$ and biomass are not reported for brevity).

The plotted curves notably show that order-based models are best suited to properly reproduce experimental trends in the case of SW. Of note, this conclusion also applies to other biomass samples, as exemplified in Tables 8 and 9, which summarize the reaction models whose use leads to the lowest RMSD values (see Sect. “Comparison between simulated and measured conversion degree profiles”). As can be seen by looking at the results from Table 8 for the KAS and Friedman methods, the smallest RMSD are obtained when selecting the F1 (SW, WS and M), F2 (SG) and F4/F5 models (SM). Alternatively, the F3 mechanism is shown to lead to the best agreement between simulated and measured results for SW, WS and M when implementing the OFW approach versus the F5 and F4 models for SM and SG, respectively. Although the RMSD values computed for the 17 different reaction mechanisms listed in Sect. “Model-free methods” are not reported herein for brevity, it should be noted that a general trend can still be drawn as far as the most suited models are concerned. Indeed, the mean RMSD values estimated when considering all the selected modeling approaches follow the sequence P$n$ > A$n$ > D > R > F.

**Table 8:** Reaction models identified as the best suited for SW, WS, SM, M and SG, considering the KAS, OFW and Friedman models

Feedstock	SW	WS	SM	M	SG
*KAS*					
Reaction model	F1	F1	F4	F1	F2
RMSD/%	2.38	3.15	1.77	3.90	3.62
${A}_{\mathrm{\alpha }}$/s^−1^ ($\alpha$=20%)	7.4E + 05	5.2E + 05	2.7E + 06	3.3E + 04	8.1E + 04
${A}_{\mathrm{\alpha }}$/s^−1^ ($\alpha$=50%)	1.8E + 07	1.2E + 07	5.1E + 07	1.1E + 06	4.6E + 06
${A}_{\mathrm{\alpha }}$/s^−1^ ($\alpha$=80%)	1.1E + 08	1.4E + 11	5.5E + 11	1.0E + 07	1.4E + 08
*OFW*					
Reaction model	F3	F3	F5	F3	F4
RMSD/%	3.50	2.90	2.50	3.51	2.75
${A}_{\mathrm{\alpha }}$/s^−1^ ($\alpha$=20%)	3.4E + 06	2.5E + 06	1.0E + 07	2.1E + 05	4.7E + 05
${A}_{\mathrm{\alpha }}$/s^−1^ ($\alpha$=50%)	1.2E + 08	8.4E + 07	2.5E + 08	9.6E + 06	3.8E + 07
${A}_{\mathrm{\alpha }}$/s^−1^ ($\alpha$=80%)	2.2E + 09	1.7E + 12	3.9E + 12	2.7E + 08	4.1E + 09
*Friedman*					
Reaction model	F1	F1	F5	F1	F2
RMSD/%	0.72	0.93	0.74	1.21	0.97
${A}_{\mathrm{\alpha }}$/s^−1^ ($\alpha$=20%)	9.7E + 06	1.6E + 07	3.3E + 07	5.7E + 05	1.3E + 06
${A}_{\mathrm{\alpha }}$/s^−1^ ($\alpha$=50%)	1.3E + 08	1.0E + 08	2.9E + 08	7.6E + 06	3.7E + 07
${A}_{\mathrm{\alpha }}$/s^−1^ ($\alpha$=80%)	9.9E + 08	1.5E + 11	4.3E + 12	3.5E + 07	7.8E + 07

RMSD averaged based on the results obtained with the 4 heating rates set during the experiments are provided together with pre-exponential factors inferred for $\alpha$ of 20, 50 and 80% as examples

**Table 9:** Reaction models identified as being the most suited for SW, WS, SM, M and SG considering the 1-step and 3-step Kissinger models together with the model-fitting approach from [1]

Feedstock	SW	WS	SM	M	SG
*1-step Kissinger*					
Reaction model	F1	F2	F3	F1	F1
RMSD/%	6.35	3.19	10.03	7.06	8.81
$A$/s^−1^	1.6E + 08	5.8E + 07	5.9E + 07	5.1E + 06	1.8E + 07
*3-step Kissinger*					
Reaction model	F1	F1	F3	F1	F1
RMSD/%	4.06	3.45	5.07	1.64	1.40
$A$/s^−1^ (PC1)	5.6E + 06	7.6E + 05	8.0E + 05	1.0E + 05	2.5E + 05
$A$/s^−1^ (PC2)	3.5E + 07	3.7E + 07	2.4E + 07	2.2E + 06	8.2E + 06
$A$/s^−1^ (PC3)	2.0E + 09	5.3E + 08	7.6E + 07	5.0E + 08	1.8E + 08
*Model-fitting approach from* [1]					
Exponent factor $n$	0.957	1.924	5.187	1.520	1.870
RMSD/%	4.57	4.25	2.60	3.26	2.94
$A$/s^−1^	4.9E + 04	1.7E + 07	7.6E + 06	7.6E + 04	1.2E + 05

The RMSD reported in the table were averaged based on the results obtained with the 4 heating rates set during the experiments

The fact that order-based models seem to be the most appropriate is further corroborated by the results depicted in Table 9, which show that the implementation of the F1 model allows computing the lowest RMSD for SW, M, SG when using the 1-step Kissinger method versus the F2 and F3 models for WS and SM. Regarding the 3-step Kissinger method, the most appropriate reaction models are similar to those identified when selecting the 1-step Kissinger method, except for WS, for which the lowest RMSD is calculated with the Mampel first-order mechanism. To conclude, it is noteworthy that the optimization procedure implemented to parameterize the model-fitting approach from [1] also allows identifying $n$-th order models as the most suited (with $n$ comprised between 0.957 and 5.187, as detailed in Table 9 (the other empirical exponent factors being found to be ~0 during the optimization process)). This hence corroborates the pertinence of selecting order-based models to properly simulate the pyrolysis of SW, WS, SM, M and SG. Of note, this conclusion is consistent with results issued from various studies dealing with biomass pyrolysis, in which order-based models were commonly identified as being well suited (see [27, 69, 74, 75], among others).

#### Comparison of simulated conversion degree profiles with measured ones and discussion regarding the potential strengths and weaknesses of the assessed models

**Fig. 6:**
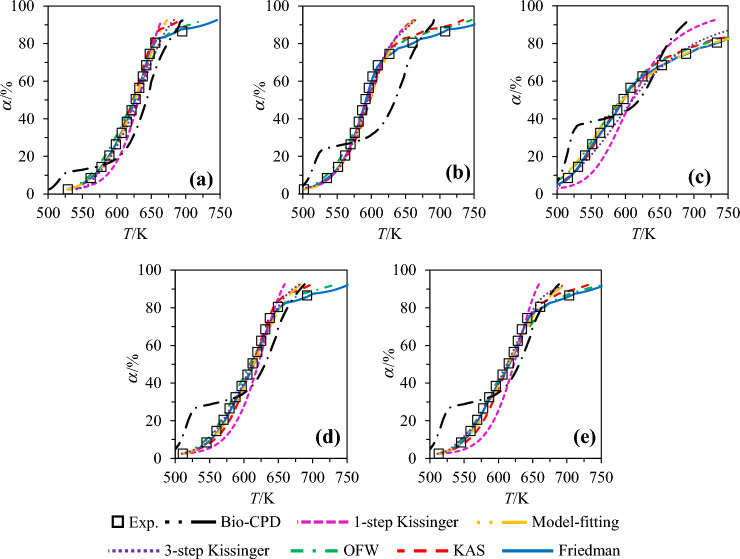
Comparison of measured and simulated conversion profiles obtained with **a** SW, **b** WS, **c** SM, **d** M and **e** SG for $\beta$ = 10 K min^−1^

The conversion degree profiles simulated using the kinetic parameters obtained from the KAS, OFW, Friedman, 1-step Kissinger, 3-step Kissinger, advanced fitting and bio-CPD models are compared with their experimental counterparts in Fig. 6 for each biomass and for a $\beta$ of 10 K min^−1^ as an example (similar trends being obtained with other heating rates). As can be seen, each tested model, with the exception of the bio-CPD, satisfactorily reproduces measured profiles, especially for 10% < $\alpha$<70%.

**Fig. 7:**
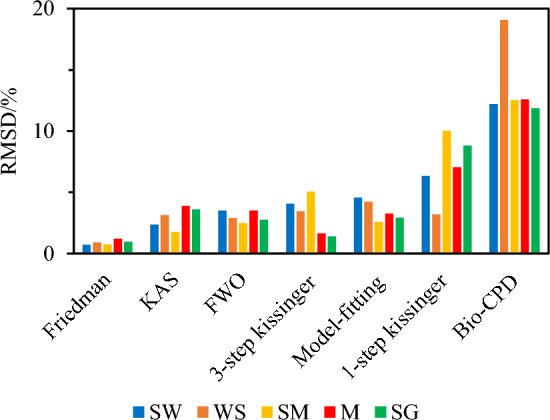
Comparison of the average calculated RMSD values for the five tested feedstocks with the 7 studied methods

That being said, and to better appreciate which model is best suited, in Fig. 7, we plotted the RMSD (averaged considering the 4 different heating rates set during the experiments) characterizing the gaps between simulated and measured data for each biomass and each modeling approach. One can thus see that the relative ability of the tested models to properly simulate measured data follows the order: Friedman > KAS ≈ OFW > 3-step Kissinger > model-fitting > 1-step Kissinger > bio-CPD.

As an isoconversional approach, the Friedman differential method is known for its attractiveness and accuracy as exemplified within the present work. Nevertheless, and although fortunately not really observed herein, this method is likely to suffer from numerical instability due to measurement noise. Indeed, one of the main drawbacks of this approach is the need for differentiation, which is prone to amplifying the data noise level, thus leading to useless data [76]. Care should therefore be taken when using the Friedman method, which especially requires processing data that is as free as possible of measurement noise, together with implementing appropriate smoothing of experimentally assessed TG signals.

As for the very popular KAS and OFW models, their use allows to effectively reproduce the measured conversion degree profiles as illustrated by their related RMSD, which are quite similar and relatively low (see Table 8 as well as Fig. 7). Here again, caution should be exercised, however, when considering the activation energies inferred using these methods (which are found to be lower herein than to those assessed with the Friedman model) since their validity can be impaired by systematic errors stemming from the assumption of constant activation energy and from the oversimplified approximation of the temperature integral [77]. This notably explains why we systematically compared and discussed the overall validity of the activation energies we inferred against previously reported data from the literature in Sect. “Estimation of activation energy values”.

Although the 1-step Kissinger approach is one of the most widely used methods for analyzing the thermal properties of pure compounds, its application to heterogeneous compounds such as biomass samples often yields unsatisfactory results [9], which is notably exemplified in Fig. 7 for $\alpha$ above 70%, when lignin begins to decompose. To address this issue, the implementation of a 3-step calculation procedure can be regarded as a relevant option as it allows accounting for the successive decomposition of the different biopolymers composing biomass. Doing so notably led to a much better agreement between theoretical and experimental profiles, as illustrated in the case of M and SG, for instance, for which the RMSD values are reduced by ⁓ 77 and ⁓ 84%, respectively, when using the 3-step approach instead of the 1-step one (see Table 9). Nevertheless, and contrary to the Friedman, KAS and OFW models, the 3-step Kissinger model is less straightforward to implement since it requires resolving the overlapped profiles of conversion rate into several independent curves by means of a deconvolution method. This process is then likely to induce uncertainties in the estimation of parameters such as the maximum peak height or the asymmetry of the ${\text{d}}\alpha /{\text{d}}T$ versus $T$ profile for some pseudo-components (see Sect. “Kissinger method”) as exemplified in the case of PC3 for WS and SM (see the uncertainties in brackets in Table 7).

Regarding the model-fitting approach from [1], it allows to derive theoretical profiles that reproduce their experimental counterparts well (see Fig. 6 as well as the RMSD reported in Table 9). Unlike other more commonly used model-fitting approaches (e.g., Coats–Redfern, Šatava–Šesták, etc.), the so-called advanced method by [1] has the advantage of not requiring setting the reaction model parameters a priori, as these latter are directly determined during the optimization procedure (see Sect. “Fitting method”). One should, moreover, note that although good matches were obtained herein between measured and simulated profiles for each feedstock, no specific effort was expended on trying to implement advanced optimization procedures (contrary to what was done in [33] for instance). A relatively simple generalized reduced gradient solver was indeed used instead, as recommended by [1]. This therefore makes the implementation of this modeling approach relatively straightforward, although we still modified the procedure initially proposed in [1] to ensure a global optimization aimed at fitting the model simultaneously to multiple data sets obtained with multiple heating rates. The activation energies inferred herein remain quite low as compared to those estimated using the above-discussed isoconversional models, however, further noting that the RMSD values computed with this fitting method are also significantly higher than those obtained when using the Friedman, KAS and OFW models.

To conclude, and contrary to the results obtained with the above models, which qualitatively and quantitatively capture the measured profiles for each biomass on the main $\alpha$ range, the theoretical curves derived from the use of the bio-CPD exhibit quite distinct features (which are typical of the profiles obtained using such a model (see [8] and references therein)), while being associated with the highest RMSD values, as shown in Fig. 7. This result can potentially be traced to the fact that the bio-CPD considers the decomposition of cellulose, hemicellulose and lignin independently. As such, it does not take into account the potential interactions between the biopolymers [78, 79] as well as the catalytic effects induced by the biomass inorganic content [4]. Furthermore, it should also be recalled that the CPD kinetic parameters were initially obtained from experimental conditions involving relatively high heating rates (of the order of 10^3^ K s^−1^), which may not be suitable for simulating data collected at a low heating rate, as previously noted in [33]. This notwithstanding, the bio-CPD still has key strengths. As examples, all it needs is to know the biochemical composition of the investigated fuel to launch a simulation, it can predict the composition and the distribution of the pyrolytic products, and its code is moreover readily available [50, 51]. To conclude, and with the exception of the results obtained with WS, for which there is greater discrepancy, the global evolution, as well as the order of magnitudes of the conversion degrees predicted by the bio-CPD, remains relatively close to experimentally assessed ones, with RMSD never exceeding 12.6%.

### Thermodynamic analysis

**Fig. 8:**
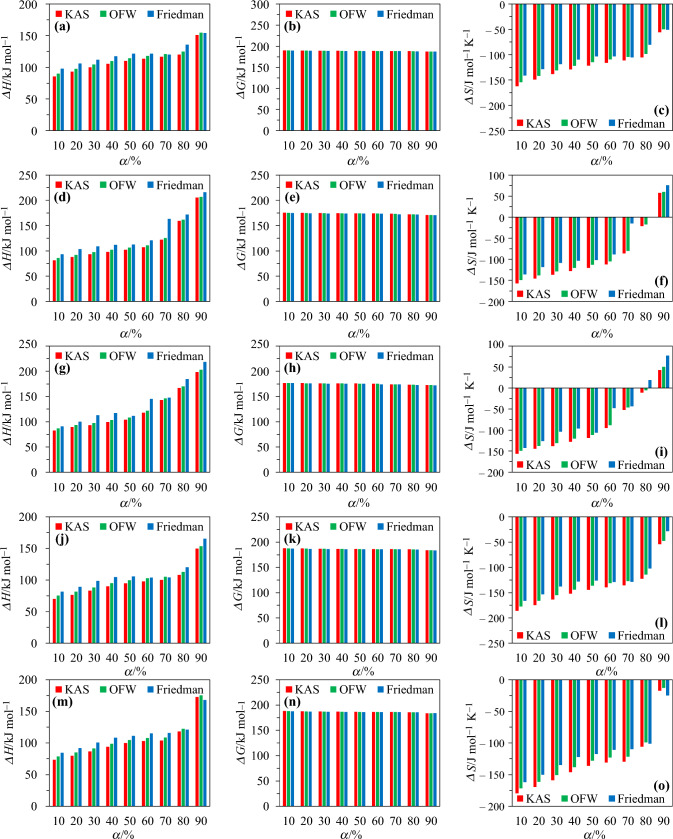
Variations of Δ*H*, Δ*G*, and Δ*S* (see Table 10) as a function of the conversion degree for SW (**a–c**), WS (**d**–**f**), SM (**g**–**i**), M (**j**–**l**) and SG (**m**–**o**)

Following the formalism adopted in [54–56], the changes of enthalpy ($\Delta H$), Gibbs free energy ($\Delta G$) and entropy ($\Delta S$) as a function of $\alpha$ for each biomass are summarized in Table 10 and plotted in Fig. 8 for convenience and clarity. As highlights, one can first note that the variations of $\Delta H$, $\Delta G$ and $\Delta S$ as a function of the conversion degree follow the same trends, regardless of the considered model (see Fig. 8). Furthermore, the $\Delta H$, $\Delta G$ and $\Delta S$ values calculated using the KAS, OFW and Friedman models are relatively close, as exemplified by the results from Table 10. The mean deviation between the $\Delta H$ values calculated using the KAS and OFW models is indeed ⁓ 4% versus ⁓ 11% when comparing the results issued from the implementation of the KAS and Friedman methods. While being consistent with what would be anticipated since the kinetic parameters inferred using these modeling approaches were quite close (see Sects. “Estimation of activation energy values” and “Simulation of conversion degree profiles”), this observation is also in line with the results issued from numerous studies using the KAS, OFW and Friedman models to compute thermodynamic parameters (see [53–56, 70, 80, 81] as examples).

**Table 10:** Thermodynamic parameters averaged over the 4 $\beta$ values (the impact of the heating rate on the calculated parameter being insignificant, as also observed in [80]) for SW, WS, SM, M and SG

*α*/%	Δ*H*_α_/kJ mol^−1^					Δ*G*_α_/kJ mol^−1^					Δ*S*_α_/J mol^−1^ K^−1^				
	SW	WS	SM	M	SG	SW	WS	SM	M	SG	SW	WS	SM	M	SG
*KAS*															
10	85.7	81.4	82.7	69.9	73.7	189.9	175.4	176.9	187.9	188.1	− 161.8	− 157.2	− 156.1	− 185.8	− 179.7
20	93.3	87.7	89.3	76.6	79.8	189.5	175.0	176.5	187.5	187.7	− 149.3	− 146.1	− 144.5	− 174.6	− 169.5
30	100.1	93.2	93.1	83.3	86.3	189.1	174.7	176.3	187.1	187.3	− 138.1	− 136.4	− 138.0	− 163.4	− 158.7
40	105.8	97.9	99.2	90.0	93.9	188.9	174.5	176.0	186.7	186.9	− 128.9	− 128.0	− 127.4	− 152.3	− 146.0
50	110.3	102.3	104.2	94.7	100.0	188.6	174.3	175.8	186.4	186.6	− 121.5	− 120.4	− 118.7	− 144.4	− 136.0
60	113.8	107.0	117.9	97.8	103.1	188.5	174.1	175.2	186.3	186.4	− 115.8	− 112.2	− 95.0	− 139.3	− 130.8
70	116.8	121.9	142.9	100.2	103.8	188.4	173.5	174.2	186.1	186.4	− 111.0	− 86.2	− 51.9	− 135.4	− 129.7
80	120.4	159.3	166.8	108.0	118.2	188.2	172.2	173.5	185.8	185.7	− 105.2	− 21.6	− 11.0	− 122.4	− 106.0
90	151.1	205.4	198.3	149.9	172.7	187.0	171.0	172.6	184.1	183.8	− 55.6	57.7	42.7	− 53.8	− 17.3
*OFW*															
10	90.2	85.8	86.7	75.1	78.7	189.7	175.1	176.6	187.6	187.7	− 154.3	− 149.4	− 149.2	− 177.1	− 171.4
20	97.8	92.1	93.3	81.7	84.8	189.3	174.8	176.3	187.2	187.4	− 141.8	− 138.3	− 137.6	− 166.0	− 161.2
30	104.6	97.5	97.2	88.3	91.2	188.9	174.5	176.1	186.8	187.0	− 130.9	− 128.7	− 130.8	− 155.0	− 150.6
40	110.1	102.2	103.3	94.9	98.6	188.7	174.3	175.8	186.4	186.6	− 121.9	− 120.6	− 120.2	− 144.1	− 138.2
50	114.6	106.4	108.3	99.6	104.6	188.5	174.1	175.6	186.2	186.3	− 114.5	− 113.1	− 111.5	− 136.3	− 128.3
60	118.1	111.0	121.7	102.7	107.8	188.3	173.9	175.0	186.0	186.2	− 109.0	− 105.1	− 88.5	− 131.1	− 123.1
70	121.0	125.4	146.2	105.1	108.6	188.2	173.3	174.1	185.9	186.1	− 104.1	− 80.1	− 46.3	− 127.2	− 121.8
80	124.6	161.6	170.0	112.9	122.5	188.0	172.1	173.4	185.5	185.5	− 98.3	− 17.5	− 5.6	− 114.4	− 99.0
90	154.8	206.8	203.0	153.8	175.4	186.9	170.9	172.5	184.0	183.7	− 49.8	60.1	50.7	− 47.4	− 13.0
*Friedman*															
10	98.2	93.3	90.6	81.7	84.4	189.2	174.7	176.4	187.2	187.4	− 141.3	− 136.2	− 142.3	− 166.1	− 161.9
20	106.2	103.4	99.9	89.3	91.5	188.8	174.3	176.0	186.7	187.0	− 128.3	− 118.5	− 126.1	− 153.4	− 150.0
30	112.1	108.9	112.9	98.7	100.7	188.6	174.0	175.4	186.2	186.5	− 118.5	− 108.9	− 103.5	− 137.8	− 134.8
40	117.7	111.9	117.2	104.6	108.3	188.3	173.9	175.2	185.9	186.1	− 109.5	− 103.7	− 96.1	− 128.0	− 122.3
50	121.6	112.7	111.6	105.7	111.2	188.2	173.8	175.4	185.9	186.0	− 103.3	− 102.2	− 105.8	− 126.1	− 117.6
60	121.6	120.6	145.2	104.0	115.2	188.2	173.5	174.2	186.0	185.8	− 103.3	− 88.5	− 47.9	− 129.1	− 111.0
70	120.3	163.1	147.8	104.3	115.8	188.2	172.1	174.1	185.9	185.8	− 105.3	− 15.0	− 43.5	− 128.6	−110.0
80	135.7	172.0	184.2	120.4	121.2	187.6	171.8	173.0	185.2	185.6	− 80.5	0.4	18.7	− 102.0	− 101.2
90	154.3	215.8	218.5	165.6	168.1	186.9	170.7	172.2	183.6	183.9	− 50.6	75.6	77.0	− 28.3	− 24.8

As far as the enthalpy change is concerned, it denotes the energy exchanged between reactants and products during the chemical reaction. As such, it is an interesting indicator of the change in the amount of heat absorbed or emitted when biomass is converted into different compounds at constant pressure. Based on the data given in Table 10, mean $\Delta H$ values in the ranges 111–121 kJ mol^−1^ for SW, 117–134 kJ mol^−1^ for WS, 122–136 kJ mol^−1^ for SM, 97–108 kJ mol^−1^ for M and 104–113 kJ mol^−1^ for SG can be estimated, depending on the kinetic model used. The fact that obtained values are positive and continuously increase with $\alpha$ (see Fig. 8) demonstrates the endothermic nature of the reaction in a nitrogen atmosphere [80], and also indicate that additional energy needs to be provided to convert the tested biomass into different products, including gas, oil or char [56]. Furthermore, the fact that the energy barrier between the activation energy and $\Delta H$ values are relatively low (⁓5.4 kJ mol^−1^ for SW, ⁓5.0 kJ mol^−1^ for WS and SM versus ⁓5.3 kJ mol^−1^ for M and SG) means that the formation of activated complex is favored and that the reaction can thus begin quickly [53]. Finally, it is noteworthy that using the KAS model, [70] found a mean $\Delta H$ of 123.07 for WS, which is close to the 117 kJ mol^−1^ value found herein. Similarly, [67] computed a discrepancy between the activation energy and the change of enthalpy of 5.0 kJ mol^−1^ when studying the pyrolysis of WS, which is in line with the value reported above [82]. Eventually estimated a mean $\Delta H$ of the order of 100 kJ mol^−1^ for woody biomass versus 111 kJ mol^−1^ in this work, thus, here again, corroborating the overall consistency of the results we obtained.

As for the change in Gibbs free energy, it is an important state function allowing to assess the degree and spontaneity of reactions while also representing the available energy of a given biomass upon combustion [56]. High $\Delta G$ values typically indicate that a large amount of energy is consumed by pyrolysis, whereas lower values conversely suggest that the desired products will be obtained with a lower energy supply. Based on the results detailed in Table 10, one can calculate a mean $\Delta G$ of ⁓ 173.6 and ⁓ 175.0 kJ mol^−1^ for WS and SM when averaging the results issued from the implementation of the three modeling approaches. On the other hand, higher values of ⁓ 188.5, ⁓ 186.2 and ⁓ 186.3 kJ mol^−1^ are assessed for SW, M and SG, thus reflecting the bioenergy potential of these feedstocks according to [53].

To conclude, the change in entropy reflects how near a system is to its own thermodynamic equilibrium. High $\Delta S$ values therefore suggest a high reactivity leading to the rapid formation of activated complex. Low activation entropy values alternatively indicate that the system, which is near its own thermodynamic equilibrium, exhibits a poor reactivity. As a consequence, few physical and chemical changes occur and the reaction takes longer to form the activated compound. As can be seen by looking at the results plotted in Fig. 8c, l and o, negative $\Delta S$ values are obtained for SW, M and SG, regardless of the conversion degree, thus indicating that the activated complex has a more organized structure as compared to the initial reactant. On the other hand, the plots obtained in the case of WS and SM (see Fig. 8f and i) exhibit increasing $\Delta S$ values, which even become positive for high conversion degrees. Such a behavior, also observed in [56], thus indicates that the activated complex becomes more disordered for $\alpha$ > 80%. The fact that the entropy changes continuously increase with $\alpha$, regardless of the biomass, finally tends to illustrate an increase of the reactivity of the samples, noting that the so-observed $\Delta S$ variations agree with the increase of the pre-exponential factors reported in Table 8.

## Conclusions

The present benchmarking analysis aimed at assessing the relative ability of 7 modeling approaches to infer kinetic parameters suitable for simulating the pyrolysis of 5 biomass types (SW, WS, SM, M and SG). Based on the results obtained, the following conclusions can be drawn:

- The Friedman model led to the best agreement between measured and simulated conversion degree profiles, followed by the KAS and OFW models. The validity of the activation energies inferred using these approaches should still be examined with caution, especially at high conversion rates, since the Friedman differential method is sensitive to measurement noise, whereas the approximations underlying the KAS and OFW approaches may induce systematic biases.
- The 1-step Kissinger model failed to properly simulate the conversion rates measured with the biomass samples tested herein, especially at high conversion degrees, at which lignin decomposes. Alternatively, the implementation of a 3-step approach accounting for the decomposition of each biopolymer allowed obtaining RMSD values very close to those assessed with the tested isoconversional models. While being one of the most physically realistic modeling approaches, the 3-step Kissinger method, however, requires resolving the overlapped conversion rate profiles into several independent curves by means of a deconvolution method, which is likely to induce uncertainties.
- The advanced model-fitting method tested in this work was demonstrated to perform well. Despite RMSD values higher than those estimated when using the isoconversional and 3-step Kissinger models, this approach has the advantage of not requiring setting the reaction model parameters a priori.
- The worst agreement between simulated and measured data was obtained when implementing the bio-CPD model. This has been traced to various factors, including the use of kinetic parameters initially validated for higher heating rates, together with the potential interactions between the biopolymers and the catalytic effects induced by inorganics in biomass, which are not considered.
- Order-based models were demonstrated to be the best suited for simulating the pyrolysis of SW, WS, SM, M and SG.
- Mean activation energies of ⁓ 121, 129, 133, 107 and 113 kJ mol^−1^ were inferred for SW, WS, SM, M and SG, respectively, while using the isoconversional models.
- The analysis of changes of enthalpy *(*$\Delta H$), Gibbs free energy ($\Delta G$) and entropy ($\Delta S$) exemplified the endothermic nature of the pyrolysis process, the reduced energy barrier between the activation energies and $\Delta H$ which favors the formation of activated complex, the bioenergy potential of SW, M and SG and the global formation of products having a more organized structure than the initial reactants.

By providing insights into how to select and parameterize particular kinetic modeling approaches, this work should be useful to researchers working on biomass pyrolysis while paving the way for complementary comparative analyses based on the use of other kinetic modeling approaches, including distributed activation energy ones, for instance.
